# Behcet’s disease and cardiovascular involvement: our experience of asymptomatic Behcet’s patients

**DOI:** 10.5830/CVJA-2014-003

**Published:** 2014-04

**Authors:** Zeynep Ulusan, Ayse Serap Karadag, Mehmet Tasar, Mehmet Kalender, Osman Tansel Darcin

**Affiliations:** Kecioren Research and Training Hospital, Ankara, Turkey; Department of Dermatology, 100.yil University Medical School, Van, Turkey; Department of Surgery, Konya Education and Research Hospital, Konya, Turkey; Department of Surgery, Konya Education and Research Hospital, Konya, Turkey; Department of Surgery, Konya Education and Research Hospital, Konya, Turkey

**Keywords:** Behcet’s disease, venous thrombosis, valvular heart disease

## Abstract

**Abstract:**

Behcet’s syndrome is a systemic inflammatory disease associated with vasculitis, and arterial, venous and cardiac disorders. Thirty-eight Behcet’s disease patients were examined prospectively with echocardiography, ultrasonography and computed tomography, and coagulation parameters were determined. Deep venous insufficiency was found in 16 patients, venous thrombosis in seven, one patient had iliac artery stenosis, three had carotid arterial intimal proliferation, two patients had aortic annulus dilatation, six had aortic valve insufficiency, and three had mitral valve insufficiency. None had coagulation defects. To decrease morbidity and mortality rates, a multidisciplinary approach is important for early diagnosis of cardiovascular involvement in Behcet’s disease.

## Abstract

Behcet’s disease is generally defined by oral and genital ulcers and uveitis. It is also known as a recurrent multisystemic and inflammatory disease. It is mostly seen in Mediterranean countries and the Far East.

The aetiology of Behcet’s disease is associated with viral, toxic, bacterial and immunological factors. It was defined in 1963 as an auto-immune disease caused by auto-antibodies against the oral mucosa. Vascular involvement is 2–7% and it is usually seen in patients between the ages of 20 and 40 years.

Behcet’s disease is a non-specific arterial and venous vasculitis.^1^-^8^ Proximal and distal anastomotic aneurysm formation after surgery is not rare one to 12 months postoperatively. Recurrent surgical interventions increase the risk of mortality and morbidity.^9^,^10^ Cardiovascular involvement in Behcet’s disease includes pericarditis, coronary arterial disease, cardiomyopathy and valvular dysfunction.^11^ The aim of this study was to report our experience of cardiovascular involvement with asymptomatic Behcet’s disease.

## Methods

From March 2008 to May 2009, 38 Behcet’s disease patients (20 women and 18 men) were prospectively analysed at the Kecioren Education and Research Hospital. International Behcet’s disease study group criteria were used for the diagnosis of Behcet’s disease in all patients.^12^ Follow up of the patients was one to 20 years (mean 10.5 years). Mean age was 37.8 years (range 33.8–41.7).

After questioning the patients on their medical history and detailed physical examination, radiological and laboratory studies were undertaken. Patients who had formerly been diagnosed with Behcet’s disease and followed up were included in the study, whereas those who were newly diagnosed were not included.

There was no history of smoking, hypertension, diabetes mellitus, rheumatic carditis or valve disease in the patients’ backgrounds. In some patients, no cardiac risk factors were detected except high levels of low-density lipoprotein cholesterol (LDL-C). No ventricular contractility disorder was detected on transthoracic electrocardiographic examinations.

Echocardiographic diameters were measured as transthoracic and two-dimensional by Prob, which can screen between 2 and 4 or 1.5 and 4.5 MHz. Volumes were measured with two and four blank images by the modified Simpson’s method. Each valve structure and its function was evaluated by Vivid 3 pro series Ge Vivid 3 echocardiography (GE Medical Systems, Milwaukee, USA).

Doppler ultrasonography was performed in B-mode and colour-mode spectral examinations with 13.5- and 9.4-MHz linear probes (Antares, Siemens AG, Medical Solutions Henkestr, Erlangen, Germany). Upper and lower extremity arterial and venous (for venous insufficiency, thrombosis, arterial stenosis and aneurysm) and carotid examinations were carried out.

Thorax and abdominal computarised tomography was performed in 5-mm sections, initially unenhanced, three to five minutes after venecontrast substance was injected into the peripheral veins, to determine abdominal and thoracic vascular structures (Simens Somatom Sensation 16 software version A50 Germany).

Blood samples were taken between 9:00 and 10:00 after an overnight fast and no viral, infectious or immunological diseases were detected. Laboratory studies were carried out on venous blood (9 units), which was centrifuged for 15 minutes at 20ºC and 4 500 rpm. It was decanted into silicone tubes (Vacutainer, Becton Dickinson, New Jersey, ABD) containing 0.105 M trisodium citrate (1 unit). Total cholesterol and triglyceride levels were detected by enzymatic methods (Roche Diagnostics, Mannheim, Germany). High-density lipoprotein cholesterol (HDL-C) levels were detected following sodium phosphotungustate–magnesium chloride precipitation. LDL-C levels were calculated according to the Friedwald formula. Leukocyte, platelet, erythrocyte, white blood cell, haematocrit and haemoglobin counts were performed by Sysmex 9000 (Roche Diagnostics) device.

Coagulation screening tests, coagulation factors, inhibitors and fibrinolysis tests were performed using the Dade Behring System (BCS, Dade Behring, Marburg, Germany) as follows:

• Prothrombin time (PT) and active partial thromboplastin time (aPTT) were measured using conventional methods and the results were saved on a coagulometer (BCS, Dade Behring).
• D-dimer levels were measured with the Plus D-dimer test (Dade Behring) method. This is a turbidometric test enriched with latex, which was developed for quantitative analysis of cross-linked fibrin degradation products in human plasma. It is done by a Dade Behring coagulation analyser device.
• Coagulation factors (F VII, F VIII, F IX, F X, F XI and F XII) were measured by coagulometric methods and *in vitro* diagnostic devices.
• The activated protein:C resistance ratio (APCR) was measured using a modified APC kit and automatic coagulometer (BCS, Dade Behring).
• In order to identify the von Willebrand factor ristocetin co-factor activity in human plasma, the thrombocyte agglutination method and in vitro diagnostic devices were used.
• Antithrombin activity was measured using a Berichom ATIII using the synthetic chromogenic substrate method.
• Protein C and S activities were measured with a coagulometric method.
• Plasminogen and antiplasmin activities were measured using a chromogenic substrate method.
• Lupus anticoagulants were identified using modified dilute Russell viper venom (LA scanning separator/LA2 conformation separator).
• Fibrinogen levels were identified using Claus methods.
• Plasminogen activator inhibitor levels were identified with activity-based functional tests.

## Results

The demographic variables and Behcet’s disease criteria are shown in Table 1. In the venous system, three patients had lower extremity deep-vein thrombosis (in one patient it was bilaterally). These three patients had ocular involvement. One patient had superior caval venous and bilaterally internal jugular venous thrombosis. Three patients had thrombosis in the vena saphena magna. Deep venous insufficiency was seen in 16 patients.

**Table 1:** Demographic variables and criteria for Behcet’s disease.

*Parameters*	*n (%)*
Age (years)	37.8 (33.8–41.7)
Gender (male/female)	24/14
Time since the diagnosis (years)	7.6 (6.1–9.1)
Recurrent oral ulcer (%)	38 (100)
Genital ulcer (%)	24 (63)
Arthralgia (%)	31 (84)
Leather findings (%)	23 (62)
Eye involvement (%)	4 (10.5)
Positive Paterji test (%)	12 (3.2)

In the arterial system, iliac artery occlusion was seen in one patient (this patient had in-stent stenosis and a history of balloon angioplasty). Three patients had carotid artery–intimal hyperplasia.

Echocardiography: left ventricular systolic/diastolic diameter was 4.2–6/4.3–6 cm, the aortic annulus was 4 cm in two patients, six patients had 2+ aortic valve insufficiency, and three patients had 1+ mitral valve insufficiency. Aortic valve involvement was 16%; 24 patients (63%) had venous insufficiency, three had deep-vein thrombosis (they all also had ocular involvement). One patient had superior vena caval and bilaterally internal jugular venous thrombosis and ocular involvement. One patient had iliac arterial thrombosis and stent history. Three patients had carotid artery intimal hyperplasia.

Table 2 shows laboratory results for coagulation and lipid parameters.

**Table 2:** Laboratory findings for coagulation and lipid parameters.

*Parameters*	*Mean value*	*Minimum value*	*Maximum value*	*Normal values*
Total cholesterol (mg/dl)	167	149	227	0–200
Low-density lipoprotein cholesterol (mg/dl)	148	69	185	0–130
High-density lipoprotein cholesterol (mg/dl)	47	41	52	0–60
Very low-density lipoprotein cholesterol (mg/dl)	22	18	25	5–40
Triglycerides (mg/dl)	104	87	121	0–150
Fibrinogen (mg/dl)	433	383	483	200–400
D-dimer (μg/dl)	215	142	289	125–375
Protein-S (%)	108	81	134	70–150
Protein-C (%)	103	90	116	60–125
Antithrombin 3 (%)	45	36	56	75–125
PTT (sn)	12.2	11.5	12.9	10–14
APTT (sn)	32	29	35	31–40
Vitamin B_12_ (pg/dl)	533	414	653	200–950
Folate (ng/dl)	7.8	4.8	10.7	3–17
Homocysteine (mmol/l)	12.4	9.5	15.2	5–15
Factor 5 (%)	89.6	78.5	100.7	79–121

## Discussion

Behcet’s disease is a multi-systemic inflammatory disorder. Autoimmune factors are involved in its aetiology. Immune fluorescence studies revealed IgM, IgG and β1 globulin on the vascular endothelial walls and the serum contained increased amounts of IgD, IgG, IgM, C1, C2, C3, C4 and immune complexes. The increased prevalence of the HLA-B5 tissue gene group suggests a genetic role as aetiological factor.^13^-^15^

Vascular Behcet’s disease occurs more frequently in patients with ocular involvement. Behcet’s disease is a non-specific vasculitis involving both veins and arteries. Infiltration of lymphocytes, mononuclear cells and mast cells can be observed around the blood vessels, causing endothelial swelling and fibrinoid degeneration. Venous system involvement is mostly seen as thrombosis and/or varicosis.

Arterial system involvement is rare and may be seen as arterial thrombosis and aneurysm. Two-thirds of arterial involvement cases consist of aneurysm and the remaining one-third are occlusive arteritis. Vascular invasive interventions mostly end up with relapsing aneurysms at either the proximal or distal ends of grafts. Postoperative aneurysm progress ranges from one to 12 months. Even catheterisation for vascular imaging may cause aneurysms, and recurrent surgery increases the mortality rate and morbidity in patients with this disease.

In patients with Behcet’s disease, cardiac involvement can be seen as pericarditis, acute myocardial infarction and cardiomyopathy. The involvement of heart valves is rare and is reported as case reports in the literature.^16^

Behcet’s disease vascular involvement increases the risk for mortality. Serious vascular complications including ischaemic cerebrovascular events, ischaemic bowel perforation, Budd-Chiari syndrome and perforation of aneurysms occur in 8% of patients and cause death in most patients. The most common vascular event is lower-extremity venous thrombosis. Thrombosis impairing venous return to the great vessels causes superior vena cava syndrome and Budd-Chiari syndrome.^16^,^17^ Dural venous thrombosis leads to intracranial hypertension.^18^,^19^

Patients with Behcet’s disease have a tendency to thrombosis because of an imbalance between procoagulant and anticoagulant factors. Lee *et al.* reported 90% of the 171 patients in one study and 3% in another had thrombosis.^20^ Antithrombin 3, protein C and protein S deficiencies increase susceptibility to hypercoagulation. Prothrombin G20210A polymorphism and factor V Leiden mutation (506Arg/gln) have been observed in idiopathic deep-vein thrombosis.^16^,^17^,^21^

Superior and inferior vena cava thrombosis have been reported at 9 and 2.5%, respectively.^11^ In our study we observed lowerextremity deep-vein thrombosis in three cases. One patient had a thrombosis in the superior vena cava and internal jugular vein and three had vena saphena magna thrombosis.

Most commonly, arterial aneurysms are seen in the abdominal aorta, and in decreasing frequency, in the femoral artery, popliteal artery and pulmonary artery, respectively. In our study we did not detect any cases of aneurysm. We did find arterial occlusion in three (1%) patients, one of which was occlusion at the iliac level. In one study, prevalence was reported as 2.2% in a series including 450 cases.^22^

Arterial involvement is most common in the form of aneurysm and pseudo-aneurysm formation, and occasionally arterial stenosis. Arterial occlusion can result in organ failure and sometimes causes infarct. The involvement of major arteries usually occurs after an average of 5.8 years and mainly involves lower-extremity arteries, and rarely upper-extremity arteries. Ranked in ascending order of prevalence, the right pulmonary artery, femoral, popliteal, subclavian and carotid arteries are involved.

Histopathological studies reveal non-specific vasculitis. Mononuclear and neutrophilic infiltration, endothelial proliferation, destruction of the elastic lamina, fibrinoid necrosis and thrombus are pathological evidence of the disorder seen at the tissue level. Combined treatment with corticosteroids, anticoagulants and immunosuppressive therapy is necessary.^22^-^24^

In a study by Hong *et al.*, it was reported that carotid arterial intimal thickness increased compared to the control group, which included normal healthy subjects (carotid artery intima–media thickness in Behçet’s disease patients without significant cardiovascular involvement).^25^ Uveitis or retinal vasculitis was found to correlate with increased carotid intimal thickness.

While Balile *et al.* found increasing age and serum cholesterol levels in patients was directly proportional, with steroid use, a correlation was not found.^27^ In our study, in patients with carotid artery intimal thickening, there was no correlation between serum cholesterol levels, age and the use of steroid eye ointment, and none of the patients had retinal vasculitis.

In a study conducted in Korea, an increase in regional arterial segmental stiffness was reported and it reached statistical significance.^28^ The exact pathophysiology of heart valve involvement causing insufficiency is unclear in patients with Behcet’s disease. Inflammation causes destruction of the valve tissue and dilatation of the ascending aorta and sinus Valsalva aneurysm. Histological findings varied from normal tissue to fibrosis independent of inflammation.

Accumulation of inflammatory cells in the adventitia and media is seen in inflammatory cases. Neutrophils, lymphocytes, plasma cells, less frequently histiocytes and eosinophilic cells, and sometimes giant cells are observed. Fibrous thickening of the intima and adventitia can be traced. However, fibrous changes to the elastic membrane has also been reported.^29^ Valves with aortic regurgitation were found in six patients in our study but fibrous thickening was not observed in the structure of the valve, and the aortic annulus was normal except in two patients.

Dilated cardiomyopathy has been reported in patients with Behcet’s disease. In our study, two patients presented with increased left ventricular diameter. Heart valve involvement is rare but if present, increases the mortality rate. We believe patients should be followed up with echocardiography annually. In our study, we detected increased incidence of venous insufficiency. To lower thrombosis risk in patients with Behcet’s disease, accompanying venous insufficiency should be diagnosed and treated early.

## Conclusion

Rates of heart and vascular involvement in Behcet’s disease range widely in the literature but this involvement increases the risk of mortality and morbidity, requiring a multidisciplinary approach.
